# Restriction site-associated DNA sequencing technologies as an alternative to low-density SNP chips for genomic selection: a simulation study in layer chickens

**DOI:** 10.1186/s12864-023-09321-5

**Published:** 2023-05-19

**Authors:** Florian Herry, Frédéric Hérault, Frédéric Lecerf, Laëtitia Lagoutte, Mathilde Doublet, David Picard-Druet, Philippe Bardou, Amandine Varenne, Thierry Burlot, Pascale Le Roy, Sophie Allais

**Affiliations:** 1 NOVOGEN, 5 rue des Compagnons, Secteur du Vau Ballier, Plédran, 22960 France; 2 PEGASE, INRAE, Institut Agro, Saint-Gilles, 35590 France; 3 SIGENAE, GenPhySE, Université de Toulouse, INRA, ENVT, 24 chemin de Borde-Rouge - Auzeville Tolosane, Castanet Tolosan, 31326 France

**Keywords:** Genomic selection, Layer chicken, Low-density panel, Imputation accuracy, Genomic evaluation accuracy, NGS, Genotyping-by-sequencing

## Abstract

**Background:**

To reduce the cost of genomic selection, a low-density (LD) single nucleotide polymorphism (SNP) chip can be used in combination with imputation for genotyping selection candidates instead of using a high-density (HD) SNP chip. Next-generation sequencing (NGS) techniques have been increasingly used in livestock species but remain expensive for routine use for genomic selection. An alternative and cost-efficient solution is to use restriction site-associated DNA sequencing (RADseq) techniques to sequence only a fraction of the genome using restriction enzymes. From this perspective, use of RADseq techniques followed by an imputation step on HD chip as alternatives to LD chips for genomic selection was studied in a pure layer line.

**Results:**

Genome reduction and sequencing fragments were identified on reference genome using four restriction enzymes (EcoRI, TaqI, AvaII and PstI) and a double-digest RADseq (ddRADseq) method (TaqI-PstI). The SNPs contained in these fragments were detected from the 20X sequence data of the individuals in our population. Imputation accuracy on HD chip with these genotypes was assessed as the mean correlation between true and imputed genotypes. Several production traits were evaluated using single-step GBLUP methodology. The impact of imputation errors on the ranking of the selection candidates was assessed by comparing a genomic evaluation based on ancestry using true HD or imputed HD genotyping. The relative accuracy of genomic estimated breeding values (GEBVs) was investigated by considering the GEBVs estimated on offspring as a reference. With AvaII or PstI and ddRADseq with TaqI and PstI, more than 10 K SNPs were detected in common with the HD SNP chip, resulting in an imputation accuracy greater than 0.97. The impact of imputation errors on genomic evaluation of the breeders was reduced, with a Spearman correlation greater than 0.99. Finally, the relative accuracy of GEBVs was equivalent.

**Conclusions:**

RADseq approaches can be interesting alternatives to low-density SNP chips for genomic selection. With more than 10 K SNPs in common with the SNPs of the HD SNP chip, good imputation and genomic evaluation results can be obtained. However, with real data, heterogeneity between individuals with missing data must be considered.

## Background

Genomic selection, as described in 2001 by Meuwissen et al. [1], has been mostly implemented in layer and broiler breeding through the use of the 600 K Affymetrix® Axiom® high density (HD) genotyping array, developed by Kranis et al. in 2013 [2]. This HD SNP chip is based on single nucleotide polymorphisms (SNPs) corresponding to variations in single nucleotide bases, frequent in DNA. The principle of genomic selection is to evaluate the genomic values of the genotyped selection candidates with or without phenotypes from a reference population with phenotypes and genotypes. This allows us to choose the best breeders for one or more traits to generate offspring of the next generation.

The cost of such HD SNP chips is still a problem for all livestock species. However, it is possible to reduce the cost of genomic selection through the use of a low-density SNP chip by selecting a subset of markers from the HD SNP chip and to impute the genotypes at missing markers. This is a very common method used in many livestock species such as cattle [3–7], pigs [8–10], sheep [11–13] and poultry [14–16]. Nevertheless, depending on the number of individuals used to design the genotyping array, this method may result in a skewing of the distribution of allele frequency towards common alleles [17]. This ascertainment bias is attributable to the SNPs genotyped from the genotyping array, which may not be all representatives of the genotyped individuals. Depending on the diversity level or the population structure this can lead to biased conclusions.

In parallel to these SNP chip methods, next-generation sequencing (NGS) techniques to simultaneously detect and genotype SNPs have been increasingly used in livestock species. Nevertheless, they remain expensive to routinely use for genomic selection. An alternative and cost-efficient solution is to sequence only a fraction of the genome using restriction enzymes. This solution was first named restriction site-associated DNA sequencing (RADseq) or genotyping-by-sequencing (GBS) but now refers to a large range of techniques relying on the use of restriction enzymes to detect and genotype SNPs [18]. Consequently, the term RADseq will be used in this study to refer to the different RADseq approaches. These techniques can be categorized depending on the use of one or two restriction enzymes [18–20]. RADseq [21], GBS with one enzyme [22, 23], Reduced Representation Libraries (RRL) [24], Multiplexed Shotgun Sequencing (MSG) [25], 2bRad [26] and Genotyping by Genome Reducing and Sequencing (GGRS) [27] correspond to techniques using a single enzyme. Double-digest RADseq (ddRADseq) [28], GBS with two enzymes [29, 30] and Complexity Reduction of Polymorphic Sequences (CRoPS) [31]correspond to techniques using two enzymes.

In addition, these techniques can also differ by the presence or absence of size selection of the DNA fragments during library preparation.

These techniques were first tested on plants [23, 30, 32] with or without a reference genome and then on cattle [33], pigs [27], goats [34] and poultry [35–37]. RADseq methods also enable de novo detection of SNPs for all species, even those without a reference genome [18].

As stated by Andrews et al. [18], these techniques have several steps in common in preparing sequencing libraries. They all start with enzymatic digestion with one or two enzymes followed by ligation of adapters on both sides of the fragments obtained. Depending on the technique, adaptors can contain barcodes, short sequences of 4 to 8 nucleotides, all different from each other, allowing the identification of each sample sequenced. Size selection of DNA fragments can also occur during library preparation. Depending on the method, size selection can be performed directly or indirectly (e.g. PCR amplification limit). Finally, the sequencing depth mainly depends on the considered multiplexing. However, for a given number of individuals and a given quantity of gigabases to be sequenced, the different techniques allow sequencing at different sequencing depths (less than 5X per site per individual with GBS and MSG methods to more than 20X per site per individual with initial RADseq) of a fraction of the genome [27]. It is possible to increase sequencing depth and thus the quality of genotypes, but this will increase sequencing costs per individual. For outbred populations such as livestock species and with low sequencing depth, these techniques are not easily applicable for SNP identification and genotyping due to the high level of heterozygosity and phase ambiguity in the haplotypes. This leads to many missing genotypes at a specific locus for different individuals and introduces variability between individuals. Consequently, one of the major drawbacks of these techniques is the management of missing data and variability between individuals. Many studies have shown the possibility of accurately imputing these missing data to reduce variability between individuals in plants [32, 38–40] and in cattle [41]. Finally, after having accurately handled this variability between individuals, SNPs in common with RADseq methods and genotyping arrays can be identified. In Torkamanek and Belzile [39], only 2,975 SNPs were in common with the 42,508 SNPs of the soy SNP chip. These SNPs obtained with RADseq methods were used for genomic selection. This was mainly implemented in plants [32, 38, 40] due to the lower level of heterogeneity and phase ambiguity in the haplotypes of the different species studied compared to those of livestock species. Thus, the implementation of RADseq methods for genomic selection in livestock species is far from routinely used. To our knowledge, there are few publications focusing on simulated data, illustrating the potential of RADseq methods for genomic selection in livestock species [42–44].

To date, initial RADseq [35], GGRS [36, 37], and double digest GBS [45] have been successfully used in poultry to detect and genotype SNPs. However, library preparation for the initial RADseq protocol with only one enzyme is rather complex, labour intensive and expensive. In addition, the random shearing step before size selection of the fragment introduces variability in the fragments obtainable for different individuals. Concerning the double digest GBS, the protocol is simpler, but there is also variability in the different sequenced fragments since there is no size selection of the fragments before the PCR step. Conversely, simplification of the protocol, design of adapters and barcodes and the removal of several clean-up steps to reduce the variation in fragment number between individuals make GGRS a simple and highly reproducible method [27]. This is also true for ddRADseq [28]. Thus, focusing on true HD genotyping and on real and simulated sequence data on a pure layer line, the first objective was to simulate GGRS and ddRADseq approaches and to identify the SNPs in common between those obtained from HD SNP chips and from the two different RADseq approaches. Based on these SNPs, the second objective was to impute the genotypes at missing markers to return to the genotypes obtained with the HD SNP chip. Finally, the third objective was to investigate the impact of imputation errors on genomic evaluation.

## Methods

All methods are described in accordance with ARRIVE guidelines (https://arriveguidelines.org) for the reporting of animal experiments.

### Animals

**Fig. 1:**
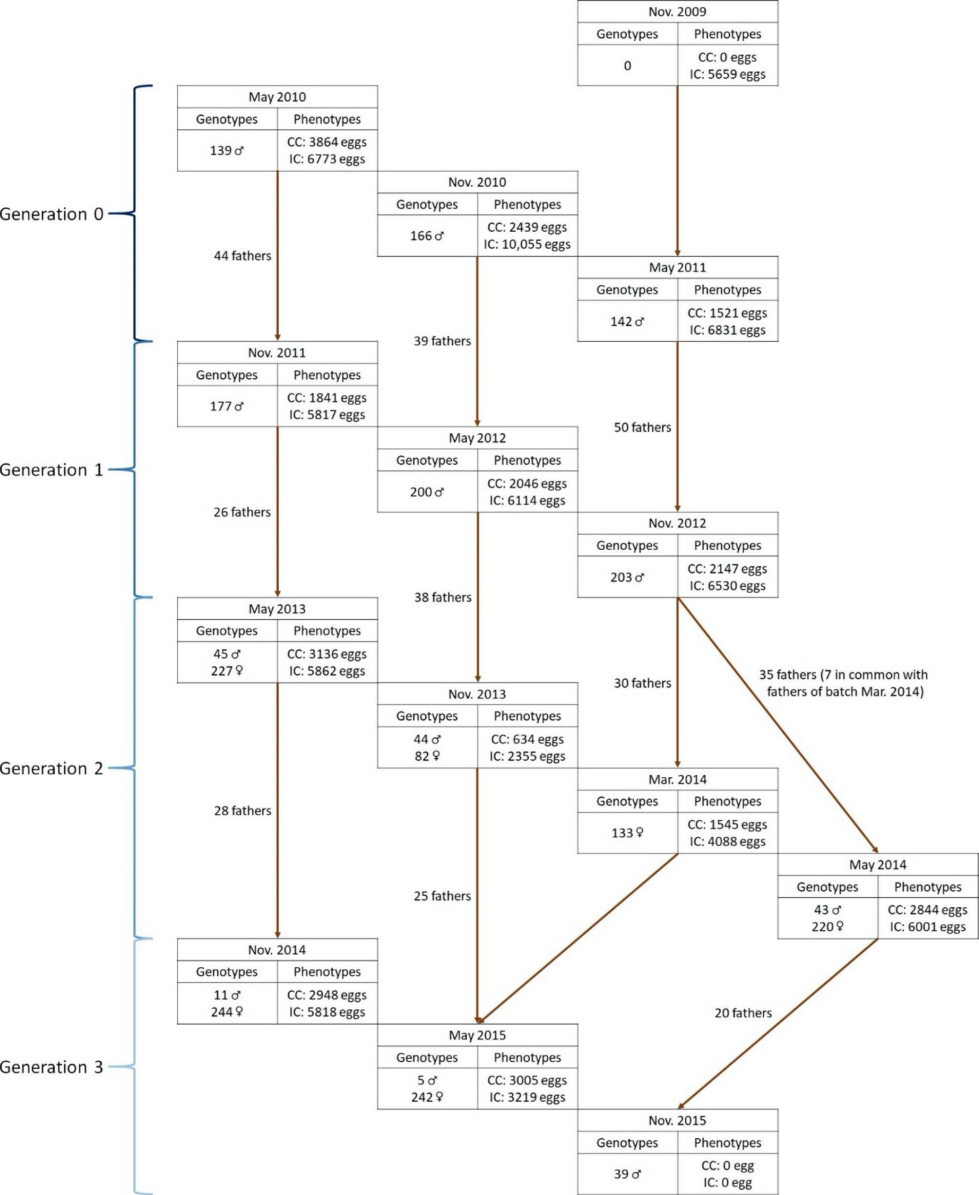
Population structure of the Rhode Island line

All animals studied consisted of a commercial pure line of Rhode Island laying hens and as detailed in Herry et al. [16]. This line was created and selected by Novogen (Plédran, France). The population comprised 21,475 chickens distributed over four generations. Each generation was represented by three batches with the breeding of a new batch every six months from 2010 to 2015 (Fig. 1).

### Genotyping

A total of 2,370 animals were genotyped for 580,961 SNPs using the 600 K Affymetrix® Axiom® HD genotyping array [2]. Genotyping acquisition is detailed in Herry et al. [16].

In accordance with the fifth annotation release of the Gallus gallus genome [46], these SNPs were distributed on macrochromosomes (1 to 5), intermediate chromosomes (6 to 10), microchromosomes (11 to 28 and 33), one linkage group (LGE64), two sexual chromosomes Z and W, and a group of 3,724 SNPs with unknown locations.

Genotypes were filtered in six successive steps (Table 1) with Plink V1.9 [47], including individual call rate (< 95%), MAF (< 0.05), SNP call rate (< 95%) and Hardy-Weinberg equilibrium (P < 10^− 4^). SNPs with unknown locations, located on linkage group LGE64 or on sexual chromosome W, were not included to assure consistency with sequence data used in this study. SNPs located on chromosomes 16 and 33 were also removed since some enzymes used in this study did not detect more than 2 SNPs in common with the SNPs of the HD SNP chip, thus preventing the possibility of imputing these chromosomes. Pedigree incompatibility problems were also checked. Finally, 300,028 SNPs and 2,362 individuals were used in this study.

**Table 1:** Summary of the different steps of quality control

Genotypes filtration	RI Line
Individual Call Rate (< 95%)	8
MAF (= 0)	204,122
MAF (< 0.05)	54,650
SNP Call Rate (< 95%)	7,541
Hardy-Weinberg equilibrium (P < 10^− 4^)	12,538
SNP with unknown location	1,748
SNP located on chromosome 16, 33, W or on linkage group LGE64	334
Pedigree Incompatibility problem	0
**SNP retained for analyses**	**300,028**
**Animals retained for analyses**	**2,362**

### Sequencing

Among the individuals genotyped with the HD SNP chip, 90 individuals of the first generation (G0) were also sequenced with Illumina HiSeq2000 technology with a target coverage of 20X at the Genomics and Transcriptomics platform GeT-PlaGe (Toulouse, France). The sequenced individuals were chosen to best represent the haplotype diversity of the chickens of the first generation (G0). Haplotype diversity was assessed by clustering on a genomic relationship matrix. Among these 90 individuals, 53 individuals were breeders of the second generation (G1), and 37 were collaterals of the breeders.

Data were aligned to the fifth annotation release of the chicken reference genome using the Burrows-Wheeler Aligner V0.7.15 [48] with default parameters for paired-end alignment. SNP calling was performed using GATK V3.7 [49] with default parameters. After applying the GATK hard filters (“FS > 60.0”, “QD < 2.0”, “MQ < 40.0”, “MQRankSum < -12.5”, “ReadPosRankSum < -8.0” and “SOR > 3.0”), 8,213,876 SNPs remained, distributed on chromosomes 1 to 28, 33 and sexual chromosome Z. The 90 whole-genome sequenced individuals were used as references to impute up to the sequence the HD genotyping of the 357 remaining individuals of the first generation (G0) and the 580 individuals of the second generation (G1). FImpute V3 [50] was used to impute these 937 individuals.

Previously, imputation tests were carried out on 90 individuals, which were sequenced at 20X and genotyped on the HD chip. These individuals were selected as representative of the genetic diversity of the line. A reference population of 50 individuals was established. Then, cross-validation tests were produced by successively drawing 8 groups of 5 individuals from the 40 remaining individuals. For each draw, a Pearson correlation was calculated between 20X sequences and imputed sequences. We obtained an average correlation of 0.98, indicating a very good imputation accuracy for HD genotyping up to the sequence level (unpublished results – personal data).

### Enzyme selection and simulations of GGRS and ddRADseq

Four distinct restriction enzymes were used to simulate in silico digestion of DNA. Consistent with Liao et al. [36] and Pértille et al. [37], two different enzymes, AvaII and PstI, were used to digest the DNA. In addition, EcoRI and TaqI were suggested by the Genomics and Transcriptomics platform GeT-PlaGe. The different sequence patterns and their sensitivity to methylation are described in Table 2.

**Table 2:** Summary of the different restriction enzymes used

Enzyme	Recognition sequence	Methylation sensitivity
AvaII	GGWCC	CpG and *dcm* methylation
EcoRI	GAATTC	CpG methylation
PstI	CTGCAG	Not sensitive
TaqI	TCGA	*dam* methylation

W denotes A or T

In addition, double digestion of DNA was simulated by simultaneously using TaqI and PstI. In silico digestion of DNA with these different enzymes was realized using R and the Bioconductor packages [51]: Biostrings [52], BSgenome.Ggallus.UCSC.galGal5 [53], reshape2 [54], scales [55] and CRAN packages : plyr [56] and ggplot2 [57]. The R script was used to identify all restriction sites on the chicken reference genome according to the enzyme used. The number of DNA fragments was counted along with the size of the fragments. Concerning the double digestion of DNA with TaqI and PstI, the R script identified all fragments obtained by the action of the two enzymes. A fragment between two restriction sites of the same enzyme cannot be used in the ddRADseq method. Fragments ranging from 200 to 500 bp were selected since they were identified as the appropriate length for sequencing fragments with the HiSeq Illumina sequencing system [58]. From the reduced list of fragments and to simulate paired-end sequencing, windows of 150 bp after the start position of the restriction site and 150 bp before the start position of a second restriction site were selected. A bed file was then created containing, for each fragment ranging from 200 to 500 bp, two sequences of 150 bp obtained using paired-end sequencing. This bed file was used with Plink to extract from the 1,027 imputed sequenced individuals all SNPs located on the 150 bp windows according to their physical positions.

Finally, among the list of SNPs extracted from the 1,027 imputed sequenced individuals, the SNPs in common with the HD genotyping after quality control were identified. The HD genotyping of the 1,027 individuals was then reduced to the SNPs previously identified, thus allowing simulation of GGRS and ddRADseq approaches.

### Imputation accuracy

In this study, the selection candidates were 580 individuals of the second generation (G1) with simulated low-density genotyping obtained through GGRS and ddRADseq simulations. Based on shared SNPs between RADseq approaches and HD SNP chips, these candidates were imputed from the HD genotyping of the 447 individuals of the first generation (G0). The selection candidates were directly related to the 447 individuals of the first generation, the fathers or the fathers’ half-brothers of the selection candidates.

For each simulated RADseq approach, imputation accuracy was estimated as the mean correlation between true and imputed HD genotypes [16]. Correlations were calculated, SNP by SNP, for all candidates according to Pearson’s method. The mean correlation was then estimated on 300,028 correlations. In addition, mean correlations were estimated for each type of chromosome. Thus, mean correlations for each type of chromosome are presented with corresponding standard deviations, excepted for chromosome Z since there is only one chromosome Z.

### Phenotypes

The four traits studied in this paper were named according to Animal Trait Ontology for Livestock [59]. Measurements of egg weight (EW), eggshell colour (ESC), eggshell strength (ESS) and albumin height (AH) were recorded between 60 and 90 weeks of age. These measures corresponded to individual measures collected in individual cages. A total of 75,121 eggs from 7,983 birds were measured from G0 to G3.

During this period, all eggs were collected and transferred at Zootests (Ploufragan, France) to study egg quality traits. Analyses started by measuring egg weight (EW, in g). A Minolta Chroma Meter was used to estimate three eggshell traits: redness (a*), yellowness (b*) and lightness (L*). Egg shell colour (ESC) was then calculated as $ESC = 100 - ({L}^{*} - {a}^{*}- {b}^{*})$. Next, a compression machine was used to evaluate the shell static stiffness, and eggshell strength (ESS, in N) was measured. ESS corresponded to the maximum force recorded before fracturing the shell. Finally, each egg was broken and albumen height (AH) measured using a tripod.

### Genomic evaluation strategies

One of the major points of interest for any breeder is to obtain good genomic evaluations from low-density genotyping simulated based on GGRS and ddRADseq approaches. The previous work of Herry el al. [60] showed a low impact of imputation errors on genomic evaluations of the selection candidates using low-density SNP chips with more than 3 K SNPs. Consequently, the next step was to validate the simulated in silico GGRS and ddRADseq methods by investigating the impact of imputed HD genotyping of the selection candidates on genomic evaluations.

EW, ESC, ESS and AH were evaluated using single-step GBLUP methodology [61] using BLUPF90 programs [62]. The four traits were jointly estimated according to a classical multitrait animal model. The fixed and covariable environmental effects of the model were described in [63]. A genomic evaluation “Full_HD” of the G1 selection candidates was performed using all available information: phenotypes and HD genotypes of ancestry (G0 animals), collaterals (G1 animals) and progeny (G2 and G3 animals). This evaluation allowed estimation of the relative maximum genomic breeding value of each selection candidate G1 and to compare this value to the results of the different genomic evaluations. The “Full_HD” evaluation was also used to estimate the genetic parameters of the model. The genetic and residual variance components were estimated using remlf90 [62]. After fixation, different genomic evaluations were carried out using blupf90.

The first objective was to investigate the impact of imputation errors on genomic evaluation based on ancestry. A genomic evaluation based on ancestry was performed with phenotypes of the individuals of the first generation (G0) and with HD genotyping of the 1,027 sires of the two first generations (G0 + G1). A second genomic evaluation was performed by replacing the HD genotyping of the selection candidates G1 with their imputed HD genotyping obtained using the two RADseq approaches. For each simulated GGRS and ddRADseq method and for each trait, the reordering of the selection candidates was estimated using Spearman correlations calculated between the true HD genomic estimated breeding values (GEBVs) and imputed HD GEBVs. Spearman correlations were calculated for the 67 breeders from G1 with at least 10 offspring in G2. They were also calculated for the top 150 individuals from G1 according to each trait. The percentage of individuals kept in top 150 was also estimated for each trait and each enzyme. The 67 breeders from G1 were not all included in the top 150 individuals from G1 because the 67 breeders were selected according to a multitrait index, whereas the top 150 individuals were different according to the trait studied.

The second objective was to study the attainable relative accuracy with imputation. The “Full_HD” GEBVs represented the maximum relative accuracy attainable with this genomic evaluation considering all information. Thus, the results of the “Full_HD” genomic evaluation of the selection candidates G1 were compared with those from the genomic evaluation based on ancestry with imputed HD genotyping of the selection candidates G1. For each simulated RADseq method and for each trait, Pearson correlations were calculated for the 67 breeders of G1 between “Full_HD” GEBVs and GEBVs based on ancestry with imputed HD genotyping of the selection candidates G1.

## Results

### Fragment size distribution

**Fig. 2:**
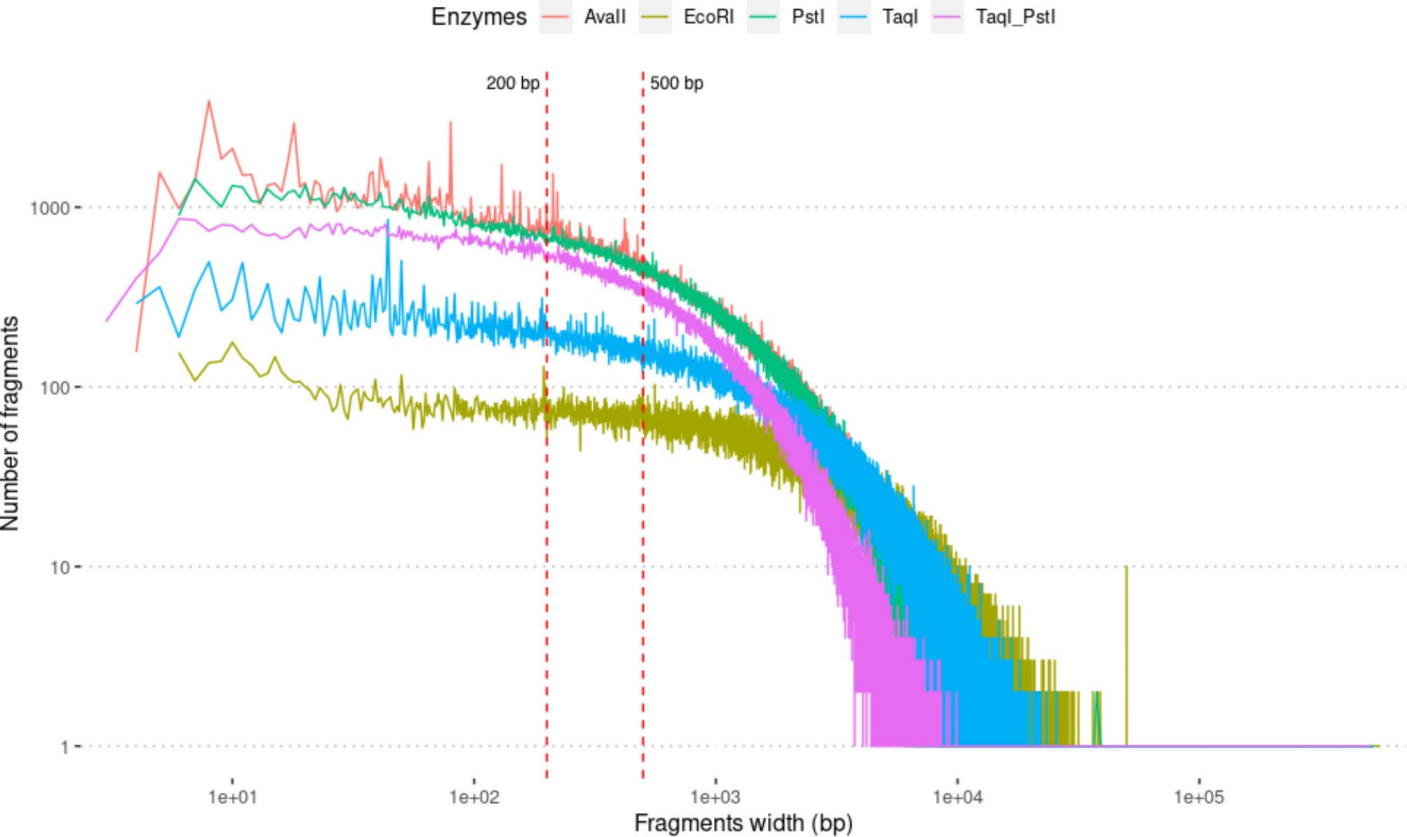
Enzymatic digestion pattern using AvaII, EcoRI, PstI, TaqI or the double association of TaqI and PstI.

The enzymatic digestion of the genome was simulated with the four different restriction enzymes as well as with double digestion with TaqI and PstI. Changes in the number of DNA fragments obtained using different enzymes according to the size of the fragments is summarized in Fig. 2. The total number of DNA fragments and the number of fragments ranging between 200 and 500 bp are presented in Table 3.

**Table 3:** Summary of the fragment number obtained, the number of SNPs detected based on the fragments and the 1,027 simulated sequences and the overlap between the HD SNP chip and SNPs detected with the different RADseq methods

	EcoRI	TaqI	TaqI + PstI	AvaII	PstI
Total Fragment number	270,629	425,312	530,105	869,482	829,382
Fragment number between 200 and 500 bp	21,267	51,163	128,823	178,980	165,804
Percentage of fragment between 200 and 500 bp	7.86%	12.03%	24.30%	20.58%	19.99%
Number of SNPs detected	46,568	122,248	318,408	470,425	427,141
Overlap between SNPs detected and HD SNP chip	1,797	4,126	11,193	12,453	14,390

In silico digestion of DNA showed, for each enzyme, a similar pattern for the changes in the number of fragments according to their length. EcoRI was the enzyme generating the fewest number of fragments (270,629), whereas PstI and AvaII generated the greatest number of fragments (829,382 and 869,482, respectively). The results were similar for the number of fragments ranging between 200 and 500 bp, with a lower number of fragments for EcoRI (21,267) and a higher number for PstI and AvaII (165,804 and 178,980, respectively). With double digestion using TaqI and PstI, a fragment obtained through the action of only one of the two enzymes cannot be considered in the ddRAD-Seq approach. The number of fragments obtained with double-digest TaqI and PstI (530,105) and after size selection of fragments ranging between 200 and 500 bp (128,823) did not correspond to the sum of the fragments obtained using TaqI and PstI separately.

Finally, PstI and AvaII and the double digestion of DNA using TaqI and PstI generated greater proportions of DNA fragments between 200 and 500 bp: 19.99%, 20.58% and 24.30%, respectively. In contrast, EcoRI produced only 7.85% fragments of interesting size.

**Fig. 3:**
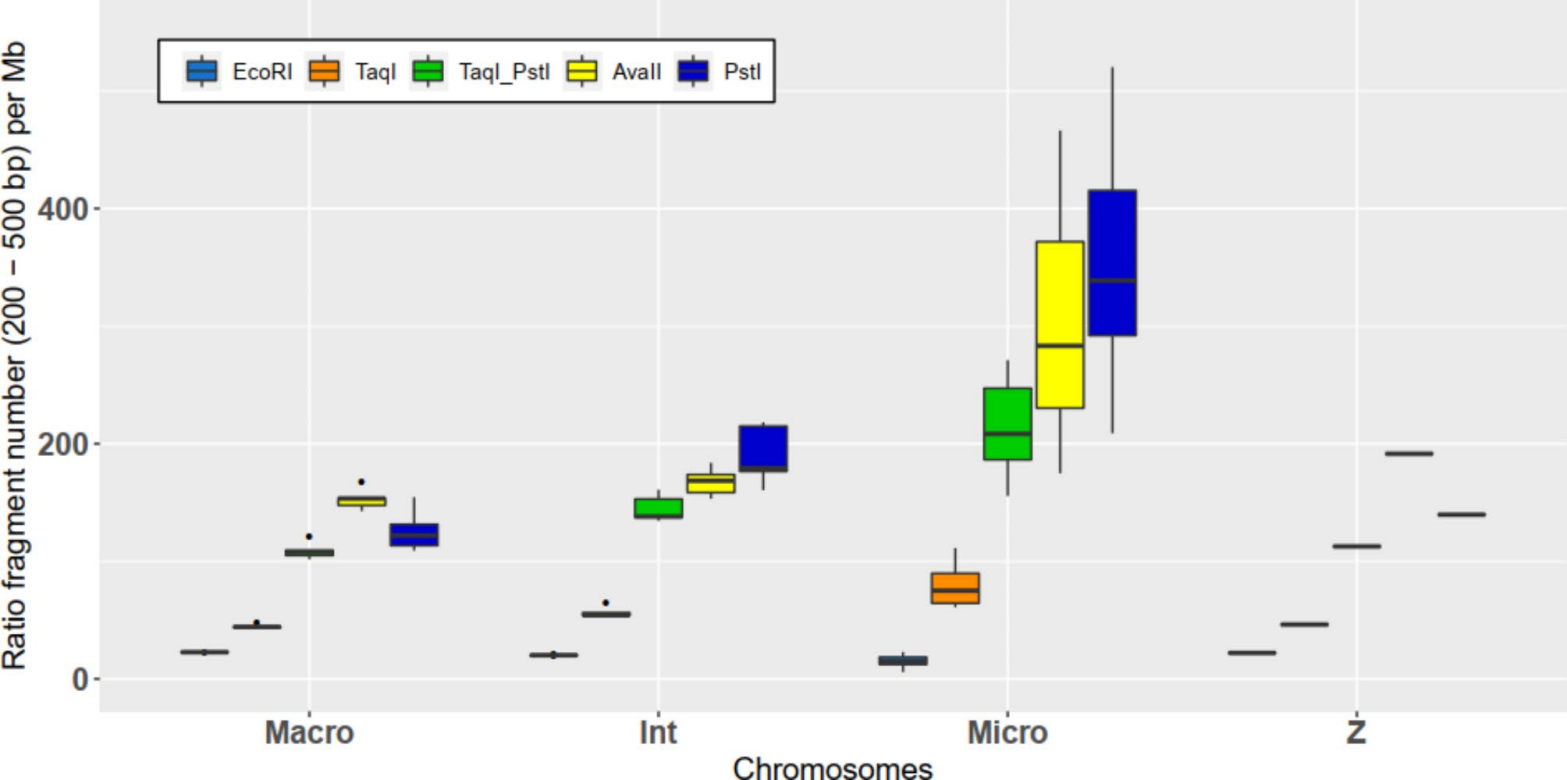
Boxplot of the number of interesting size fragments (200–500 bp) per Mb obtained for each enzyme according to the type of chromosome

For each enzyme, the number of fragments of interest (200–500 bp) per Mb (Fig. 3) was also different according to the type of chromosome. Indeed, for EcoRI, there was a decrease in the ratio from macrochromosomes (22.6 ± 0.5 s.d.) to intermediate chromosomes (20.1 ± 0.5 s.d.) to microchromosomes (15.0 ± 4.5 s.d.). For the 3 other enzymes and the double use of TaqI and PstI, the ratio increased. The most extreme case was for PstI, with an increase in the ratio from 126.1 ± 16.1 s.d. to 189.9 ± 22.8 s.d. to 346.5 ± 79.4 s.d. for macrochromosomes, intermediate chromosomes and microchromosomes, respectively. Finally, except for AvaII, the results for sexual chromosome Z were similar to those obtained for macrochromosomes.

### Overlap between a high-density SNP chip and simulated data

Based on their physical positions, fragments ranging between 200 and 500 bp were used to produce the bed file used by Plink to extract all SNPs located in the windows covered by the fragments from the 1,027 imputed sequenced individuals. This allowed us to extract 46,568 SNPs with EcoRI, 122,248 SNPs with Taq1 and 427,141 and 470,425 SNPs with PstI and AvaII, respectively. The double digestion of DNA with TaqI and PstI also enabled 318,408 SNPs to be obtained.

The overlap afforded by the number of SNPs detected with the GGRS and ddRADseq approaches and the SNPs that can be genotyped with the HD SNP chip was then compared. The number of SNPs in common was quite reduced for EcoRI, with only 1,797 SNPs. For TaqI, AvaII and PstI, the number of SNPs increased from 4,126 to 12,453 and to 14,390 SNPs, respectively. The number of SNPs in common between ddRADseq and the HD SNP chip was 11,193 SNPs. Finally, among all the SNPs detected with the GGRS and ddRADseq approaches, the proportion of SNPs in common with the HD SNP chip was 2.65% and 3.86% for AvaII and EcoRI, respectively.

### Distribution of SNP on chromosomes

**Fig. 4:**
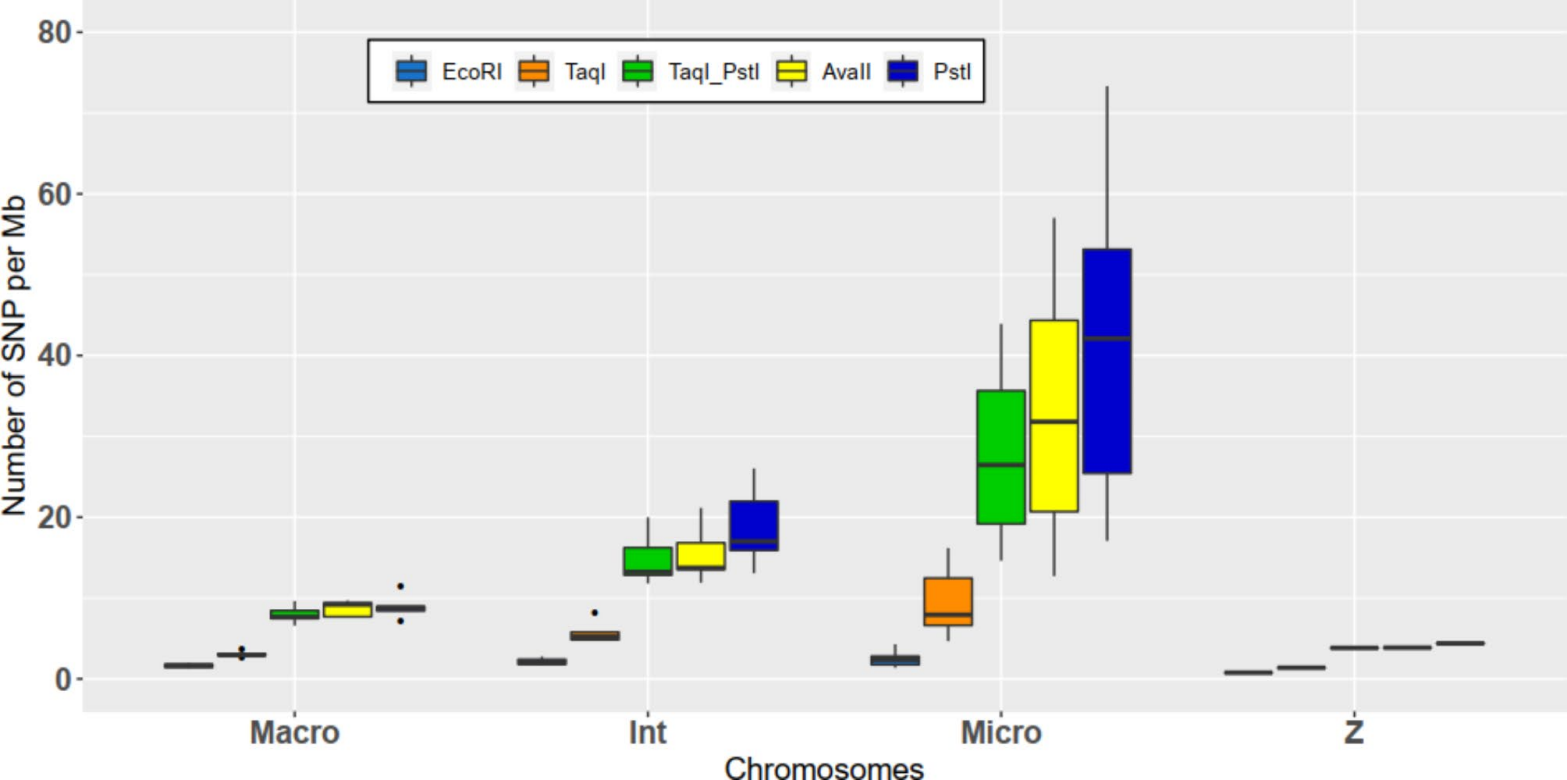
Ratio number of common SNP. Mb^− 1^ depending on the type of chromosome for each enzyme

Before studying imputation accuracy based on the SNPs in common between the HD SNP chip and those detected using RADseq methods, the distribution of these SNPs according to the type of chromosome was studied (Fig. 4). From macrochromosomes to microchromosomes, the ratio of SNPs per Mb was rather stable for EcoRI, slightly increasing from 1.6 ± 0.2 SNP. Mb^− 1^ to 2.3 ± 0.7 SNP. Mb^− 1^. However, for the other enzymes and the double digestion of DNA with TaqI and PstI, the results showed an increase in the ratio going from macrochromosomes to microchromosomes. Indeed, for TaqI and PstI, the ratio increases from 3.0 ± 0.3 SNP. Mb^− 1^ and 8.9 ± 1.4 SNP. Mb^− 1^ to 9.4 ± 3.5 SNP.Mb^− 1^ and 42.6 ± 16.3 SNP. Mb^− 1^. The ratios for double digestion with TaqI and PstI were intermediate to the ratios for TaqI and PstI. The sexual chromosome Z in particular showed a lower ratio than those observed for the other type of chromosome for each enzyme tested. The ratio was only 0.8 SNP. Mb^− 1^ for EcoRI and 4.4 SNP. Mb^− 1^ for PstI.

### Estimation of the impact of mutations on the generation of restriction fragments

We examined the impact of mutations that create new restriction sites or destroy existing ones on the Taq1/Pst1 enzyme pair to assess the impact of these creations or destructions. We determined the number of sites destroyed or created for each enzyme using genotyping data based on 20X sequencing: 57,596 sites destroyed, and 55,937 sites created for Pst1 and 32,692 destroyed and 29,482 created for Taq1. This information about restriction site creation or destruction was then combined with all previously identified site positions. We obtained a list of 129,648 restriction fragments and compared it to the previous list of 130,187 fragments without including mutations by reusing the conditions for obtaining a restriction fragment (i.e. presence of both sites in this example and size greater than 150 bp and less than 500 bp): 105,529 fragments are shared by both lists, and 24,938 and 24,389 fragments are unique to the lists without and with mutations, respectively. Finally, we extracted the SNPs potentially present in these new fragments to obtain a total of 321,090 SNPs with 10,978 SNPs shared by the HD DNA chip, compared to the previously identified 318,408 SNPs and 11,193 SNPs shared by the HD chip without mutation inclusion. When the SNP lists with and without mutations are compared, there are 227,851 common SNPs in total and 8,363 common SNPs with the HD chip. With or without considering mutations and counting common SNPs with the HD chip, we observe that 3/4 of the SNPs (8,363) are identical (the difference being explained by the difference in predicted enzyme fragments). The presence of mutations in the restriction sites does not affect the total number of predicted SNPs common with the HD chip (11,193 SNPs and 10,978 SNPs without and with mutations accounted for, respectively).

### Imputation accuracy

The imputation accuracy of the 580 selection candidates of the second generation (G1) from low-density genotyping obtained with the RADseq approaches to HD genotyping was studied for each enzyme used. These individuals were imputed from the HD genotypes of the 447 individuals of the first generation (G0).

The mean correlation between true and imputed HD genotyping was 0.7906 from 1,797 SNPs with EcoRI, 0.9121 from 4,126 SNPs with TaqI, 0.9691 from 11,193 SNPs with TaqI and PstI, 0.9699 from 12,453 SNPs with AvaII and 0.9735 from 14,390 SNPs with PstI (Table 4). There was an increase in mean correlations with an increase in the number of SNPs used to impute the selection candidates.

**Table 4:** Summary of the mean correlations of true and imputed genotypes obtained for the different enzymes for the 580 selection candidates of the second generation (G1)

	EcoRI	TaqI	TaqI_PstI	AvaII	PstI
Number of SNPs	1,797	4,126	11,193	12,453	14,390
Mean correlation	0.7906	0.9121	0.9691	0.9699	0.9735

**Fig. 5:**
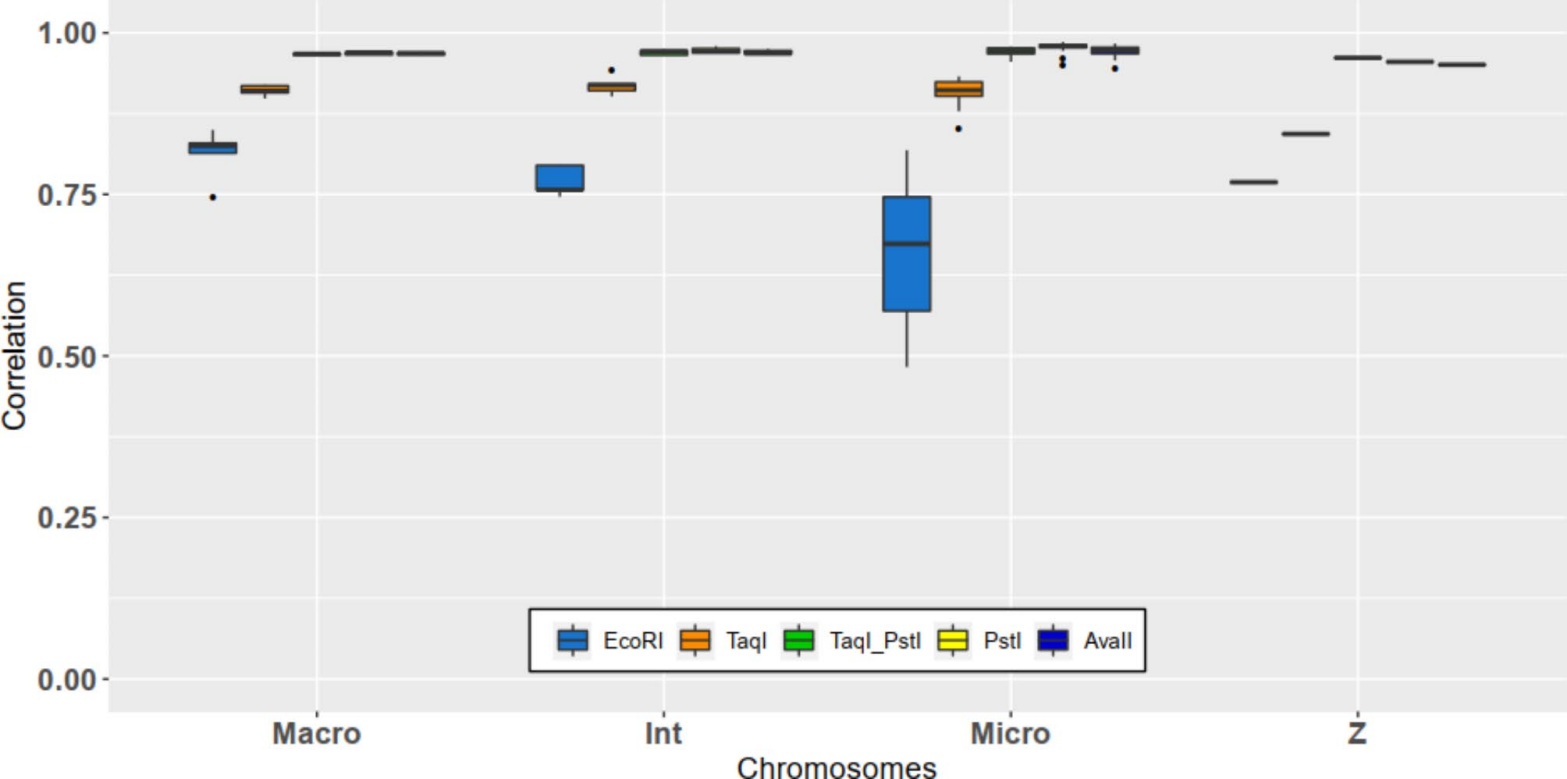
Correlations between true and imputed genotypes according to the type of chromosome for each enzyme

The influence of the type of chromosome on mean correlations for each enzyme used was also studied (Fig. 5). EcoRI presented a decrease in mean correlations going from macrochromosomes (0.81 ± 0.03) to intermediate chromosomes (0.77 ± 0.02) and to microchromosomes (0.66 ± 0.11). In contrast, the other enzymes as well as the double use of TaqI and PstI showed results that were rather stable. Indeed, for TaqI, the mean correlations were 0.91 ± 0.01, 0.92 ± 0.01 and 0.91 ± 0.02 for macrochromosomes, intermediate chromosomes and microchromosomes, respectively. Similarly, for PstI, the mean correlations were 0.97 ± 0.00, 0.97 ± 0.00 and 0.98 ± 0.01 for macrochromosomes, intermediate chromosomes and microchromosomes, respectively.

Finally, the enzyme EcoRI presented the lowest results for all types of chromosomes, and AvaII and PstI, as well as the double digestion with TaqI and PstI, showed greater results for all types of chromosomes, with slightly higher results for PstI compared to AvaII and the double digestion. However, there was no significant difference in macrochromosomes between AvaII and PstI. Likewise, there was no significant difference for intermediate and microchromosomes with AvaII and the double use of TaqI and PstI.

The correlations for sexual chromosome Z were significantly less important than the mean correlations obtained for all types of chromosomes and for each enzyme, except for EcoRI, which showed no significant difference with the mean correlation of intermediate chromosomes.

### Impact on genomic evaluations

#### Impact of imputation errors

For each enzyme used, the impact of imputation errors was studied using Spearman correlations to compare the GEBVs based on ancestry with true HD genotyping and imputed HD genotyping of the selection candidates G1. This enabled estimation of the reordering of the selection candidates. Spearman correlations were calculated for the best 150 individuals of G1 for each trait studied and for the 67 breeders of G1 having at least 10 offspring in the next generation G2 (Table 5).

**Table 5:** Spearman correlations between true HD GEBVs and imputed HD GEBVs, according to each enzyme used for egg weight (EW), eggshell colour (ESC), eggshell strength (ESS) and albumen height (AH), for genomic evaluations based on ancestry. The results are shown for the top 150 individuals for each trait and for the 67 breeders of G1

	Number of SNPs	EW		ESC		ESS		AH	
		Top150	Breeders	Top150	Breeders	Top150	Breeders	Top150	Breeders
EcoRI	1,797	0.8430	0.9450	0.6481	0.8854	0.8226	0.9255	0.8427	0.9096
TaqI	4,126	0.9388	0.9914	0.9012	0.9833	0.9501	0.9847	0.9088	0.9813
TaqI_PstI	11,193	0.9913	0.9971	0.9779	0.9938	0.9904	0.9957	0.9859	0.9973
AvaII	12,453	0.9899	0.9975	0.9737	0.9949	0.9879	0.9951	0.9867	0.9958
PstI	14,390	0.9937	0.9980	0.9781	0.9959	0.9907	0.9943	0.9848	0.9949

Concerning the top 150 individuals of G1 for each trait studied, the results were significantly lower for EcoRI than those obtained using the other enzymes. With the enzyme EcoRI, Spearman correlations were 0.8430, 0.6481, 0.8226 and 0.8427 for EW, ESC, ESS and AH, respectively. In addition, among all correlations for the different traits and enzymes, the lowest was obtained using EcoRI for ESC. For TaqI, given the standard deviations, the results were significantly greater than those obtained with EcoRI. The strongest correlations (higher than 0.97) were calculated regardless of the trait studied with ddRADseq and with AvaII or PstI. The results for these three last cases were not significantly different from each other for the different traits studied.

In addition, when focusing on the number of individuals in the Top150 that remain in the Top150 after imputation, results are consistent with Spearman correlations. The lowest numbers are obtained for EcoR1 with 132, 121, 130 and 129 individuals staying in the Top150 for EW, ESC, ESS and AH, respectively. The highest numbers are obtained for ddRAD-Seq with 146, 147, 146 and 147 individuals remaining in the Top150 for EW, ESC, ESS and AH, respectively.

Concerning the 67 breeders of G1, the results for each trait were also lower for EcoRI compared to those obtained using ddRADseq and for simple digestions with TaqI, AvaII or PstI. Spearman correlations were 0.9450, 0.8854, 0.9255 and 0.9096 for EW, ESC, ESS and AH, respectively, with the enzyme EcoRI. However, except for ESC and given the standard deviations, the results were not significantly different from the results obtained using TaqI. The results for ddRADseq or the use of AvaII or PstI were not significantly different from each other, and all resulted in correlations above 0.99, thus indicating a very reduced reordering of the 67 breeders.

Finally, with EcoRI or TaqI, based on standard deviations, the results obtained for the top 150 individuals for each trait were significantly lower than those obtained for the 67 breeders with the exception of AH with EcoRI and ESS with TaqI. For AvaII, PstI and ddRADseq, the results for the top 150 individuals for each trait were also lower than those obtained for the 67 breeders, but the differences were not significant.

#### Impact on the relative accuracy of genomic evaluation

The impact on the relative accuracy of genomic evaluation was studied using Pearson correlations to compare the results of the “Full_HD” genomic evaluation and the results of the different genomic evaluations based on ancestry with imputed HD genotyping of the selection candidates of G1. Pearson correlations were calculated for the 67 breeders of G1 having at least 10 offspring in the next generation G2 (Table 6). These results were compared to the Pearson correlation between true “Full_HD” GEBVs and true HD GEBVs based on ancestry for the 67 G1 breeders. This represented the maximum relative accuracy attainable with HD information.

With EcoRI, except for ESC, Pearson correlations for the different traits were all lower than those obtained with the other enzymes or the HD SNP chip. Indeed, the correlations were 0.3774, 0.3420 and 0.4261 for EW, ESS and AH, respectively. With the HD SNP chip, the correlations for EW, ESS and AH were 0.4713, 0.3940 and 0.4802, respectively. In contrast, the results were greater for ESC, with a correlation of 0.2962 with EcoRI and 0.2460 with the HD SNP chip. However, based on standard deviations, these differences were not significant. With EcoRI, the standard errors were ± 0.11 for EW and AH and ± 0.12 for ESC and ESS. Concerning the HD SNP chip, the standard errors were ± 0.11 for EW and ± 0.12 for ESC, ESS and AH.

The results from the other enzymes were closer to the maximum relative accuracy attainable with HD information, but the differences observed were not significant.

**Table 6:** Pearson correlations between true “Full_HD” GEBVs and imputed HD GEBVs based on ancestry for the 67 G1 breeders, according to each enzyme used for egg weight (EW), eggshell colour (ESC), eggshell strength (ESS) and albumen height (AH).

	Number of SNPs	EW	ESC	ESS	AH
EcoRI	1,797	0.3774	0.2962	0.3420	0.4261
TaqI	4,126	0.4476	0.2453	0.3906	0.4478
TaqI_PstI	11,193	0.4740	0.2442	0.3869	0.4684
AvaII	12,453	0.4681	0.2430	0.3859	0.4794
PstI	14,390	0.4664	0.2450	0.3953	0.4689
HD SNP chip	300,028	0.4713	0.2460	0.3940	0.4802

The line HD SNP chip corresponds to the Pearson correlation between true “Full_HD” GEBVs and true HD GEBVs based on ancestry for the 67 G1 breeders.

## Discussion

### Choice of the restriction enzyme

The key function of a good restriction enzyme for genotyping is to cut DNA into a number of fragments of appropriate size and to avoid frequently cutting DNA sequences leading to a large number of fragments that are too small [64]. The diversity of restriction enzymes in terms of fragment length, position of the cut site, GC content in their recognition site and sensitivity to methylation are factors that can impact this major key point [19]. Thus, the choice of a suitable restriction enzyme has to be consistent with the purpose of the study and the species studied.

In this study, to determine the choice of restriction enzyme, the total number of fragments to be sequenced per sample to enable sufficient overlap between SNPs detected with the different RADseq methods and SNPs on the HD SNP chip must be calculated. According to Liao et al. [36], the total number of fragments can be calculated by dividing the size of the chicken genome by the extent of linkage disequilibrium. This proved to be very variable between breeds, lines and chromosomes [65–67]. Thus, the range for useful LD (r² > 0.3) [68, 69] for the studied line was 200–250 kb. For macrochromosomes and microchromosomes, the range for useful LD was 400–450 kb and 100–150 kb, respectively. The most extreme case was for sexual chromosome Z, with range for useful LD of 850–900 kb. In addition, the International Chicken Genome Sequencing Consortium [70] showed that the size of a chromosome is inversely correlated with the recombination rate, methylation and gene density. Thus, it could be useful to densify the number of fragments on the microchromosomes. Assuming a useful LD of 0.3 and an extent of 100 kb, a total of 10,000 fragments of interesting size would be sufficient.

EcoRI generated 21,267 fragments of interesting sizes, but only 1,797 SNPs were found in common with the SNPs detected between the GGRS approach and the SNPs on the HD SNP chip. To ensure better overlap between SNPs identified using RADseq approaches and SNPs on the HD SNP chip, the total number of fragments should be multiplied by 10 or 20 to obtain 100,000 or 200,000 fragments. ddRADseq with TaqI and PstI enabled the production of 128,823 fragments and 11,193 SNPs in common with the SNPs on the HD SNP chip. AvaII and PstI produced 178,980 and 165,804 fragments of interesting size, respectively, and 12,453 and 14,390 SNPs were detected in common with the SNP on the HD SNP chip.

By looking at the distribution of the fragments (Fig. 3) and the SNPs in common with the HD SNP chip (Fig. 4) on the different types of chromosomes, the results showed that DNA digestion with TaqI to a lesser extent but mainly with AvaII and PstI or double digestion with TaqI and PstI enabled densification of the fragments and SNPs on microchromosomes. This was probably due to their GC-rich recognition sites (greater than 50%). Since microchromosomes have been shown to have a higher GC content [70] and a higher SNP density on the HD SNP chip, this may explain the differential distribution between different types of chromosomes. Conversely, EcoRI had a recognition site less rich in GC, leading to a lower number of restriction sites and a lower number of fragments of interesting size. This led to a lower number of SNPs on microchromosomes detected in common with RADseq approaches and HD SNP chips. The GC content of the recognition site of the restriction enzyme is thus of major importance when choosing the most suitable restriction enzyme for the species studied to ensure correct coverage of all chromosomes.

Finally, sensitivity to methylation must be taken into account in choosing the restriction enzyme. Indeed, if a methylation event (CpG, *dam* or *dcm*) occurs in a restriction site, the methylation-sensitive enzyme will not be able to cleave the DNA. This will lead to the creation of a longer fragment that may not be sequenced if the size exceeds 500 bp. From the enzymes used in this study, only PstI was insensitive to methylation, whereas EcoRI was sensitive to CpG methylation, TaqI to *dam* methylation and AvaII to CpG and *dcm* methylation. This sensitivity to methylation events was not simulated in this study but has to be taken into account with real data since this would lead to more variability between the reads for the different individuals.

### Management of heterogeneity between individuals

Regardless of the restriction enzyme used, a major drawback of the RADseq methods is the management of the variability between individuals. Indeed, as seen previously, sensitivity to methylations can lead to variability between reads for different individuals. Another factor that will increase the variability between individuals is the polymorphism occurring in restriction sites. Both factors can lead to allele dropout [18]. This phenomenon occurs for heterozygous individuals with a polymorphism in the restriction site, resulting in a failure to cut the DNA. The missing allele will therefore not be sequenced (null allele), heterozygous individuals will be considered homozygous for this SNP, and thus, genotyping errors will be introduced. By failing to cut the DNA, this phenomenon can also create longer fragments that will not have an interesting size for the study and that will not be sequenced. Although the goal of our study was to first assess the number of potential SNPs based on the enzymes used, and we can reasonably assume that the overall impact in terms of site creation or destruction is homogeneous across enzymes, we still wanted to assess the effect of mutations using the Taq1/Pst1 pair as an example. It turns out that the total number of SNPs obtained is comparable, and that the same number of fragments are created as are destroyed. However, it can be argued that these creations or destructions will be the source of genotyping heterogeneity. In the context of our study, where the second objective was to estimate the feasibility of a genomic evaluation using RADseq genotyping data, it is obvious that the actual raw data will be subjected to quality control (Call Rate SNPs and DP SNPs), and we can estimate that this quality control will eliminate most of these genotyping differences. Indeed, preliminary analyses of genotype comparisons obtained by 20X WGS and ddRAD-seq for the same individuals have shown that the majority of genotype differences (false homozygotes and frequency inversions) potentially due to the allele drop out phenomenon are eliminated by applying a SNP call rate and SNP depth filter (results in submission). Furthermore, the imputation phase can correct potential genotyping errors.

Finally, the read depth variability between loci is a factor that can influence the quality of the reads and thus the quality of the genotypes [19]. The read depth is a factor that strongly impacts the number of variants that can be detected after filtering the reads. In addition, with only one read, it is impossible to correctly call heterozygous individuals since the read informs just one allele. With two reads, it is possible to call heterozygous individuals correctly, but the probability of reading the same allele is 50%, thus leading to inaccurate calling. Gorjanc et al. [44] showed that the calling of a heterozygous individual from $n$ sequence reads can be represented by $n$ draws from a Bernoulli distribution with a probability of $1-\left(\frac{2}{{2}^{n}}\right)$. Read depth variability can be created during PCR amplification with the preferential amplification of fragments rich in GC content or with the preferential amplification of short fragments compared to long fragments [18].

**Fig. 6:**
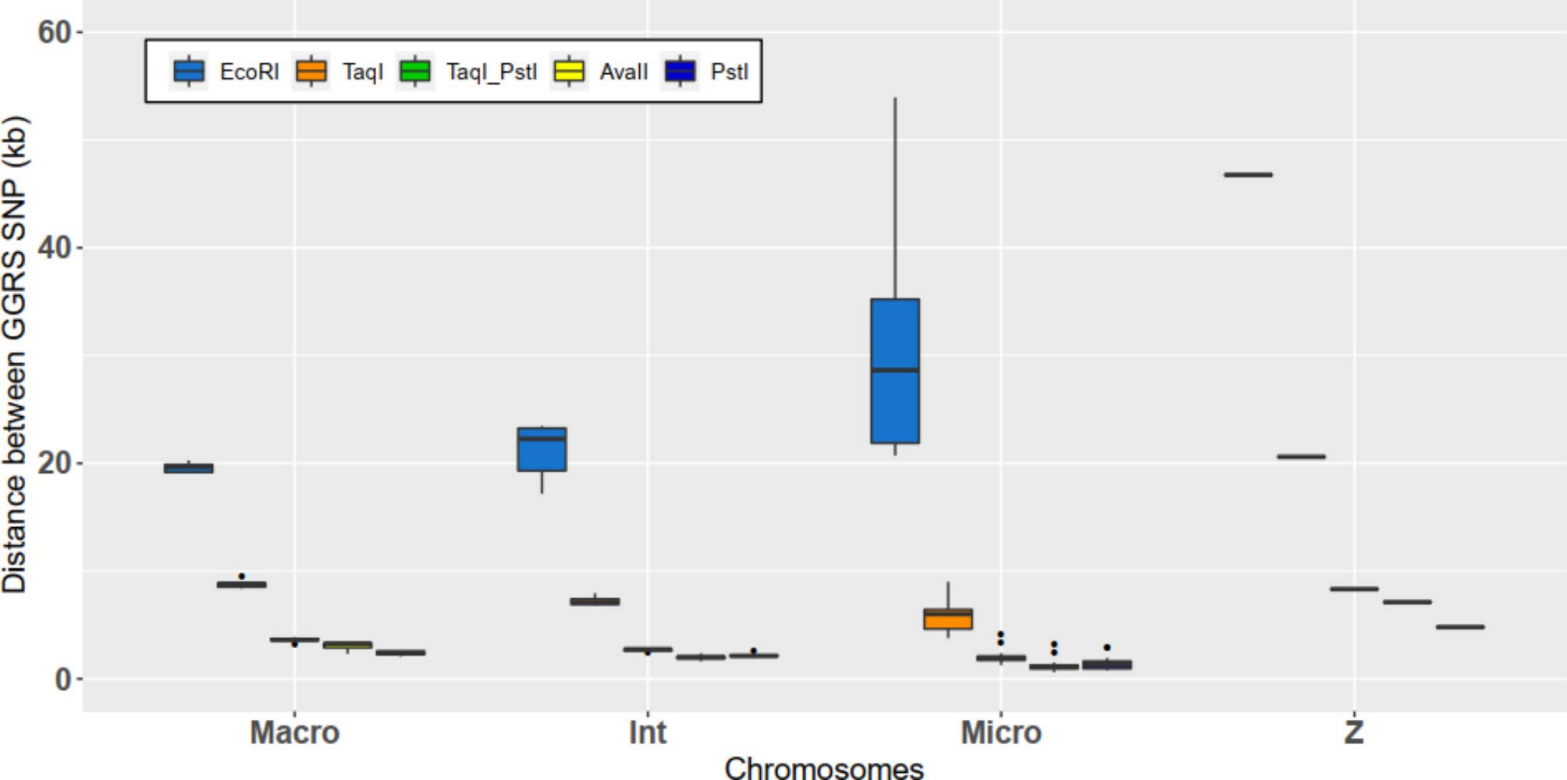
Distance between SNPs detected with GGRS and ddRADseq approaches (in kb) according to the type of chromosome for each enzyme

However, many studies have shown that this variability can be handled without a large loss of accuracy. Brouard et al. [41] studied raw reads with different levels of read depth in bovines, including or not including a filter on the call rate of SNPs and MAF. On sequences, the call rate consists of a genotype quality filter but allows management of the amount of missing data. Thanks to the imputation, they filled missing data. Based on different filters their imputation accuracy with FImpute ranged between 70% and 83.3%. The maximum accuracy was obtained for a minimum read depth of 4, a minimum call rate of 0.4 and a minimum MAF of 0.02. For the same parameters but with a minimum call rate of 0.2, their imputation accuracy was 73.3%. They also showed that filtering for a minimum MAF of 0.02 enabled slightly larger and better imputed datasets to be obtained compared with filtering for a minimum MAF of 0.05. In addition, Torkamaneh and Belzile [39] showed for Canadian soybean lines that their imputation accuracy ranged between 86% and 94% for a maximum amount of missing data of 20% and 80%, respectively. In contrast to the previous study, they noted that increasing the maximum amount of missing data (thus lowering the minimum call rate) up to 80% led to better imputed missing data than with a maximum amount of missing data of 20%. The difference in the conclusion of the two studies may be due to the shorter size of the haplotype and the lower LD extent of cattle compared to those of soybean. Indeed, the average distance for having an LD extent corresponding to half of its maximum value was between 75 kb and 150 kb for wild or cultivated soybeans, but less than 10 kb for cattle. The chicken LD extent for the line studied was between 250 kb and 300 kb. In addition, as seen in Fig. 6, for each enzyme, the whole SNPs detected with different in silico GGRS and ddRADseq approaches were separated by a distance much less than 250 kb. Indeed, for EcoRI, the distances between SNPs were 19.63 ± 0.43 kb, 21.09 ± 2.46 kb, 30.32 ± 9.47 kb and 46.76 kb for macrochromosomes, intermediate chromosomes, microchromosomes and sexual chromosome Z, respectively. For PstI, the distances were 2.38 ± 0.20 kb, 2.18 ± 0.18 kb, 1.45 ± 0.63 kb and 4.80 kb for macrochromosomes, intermediate chromosomes, microchromosomes and sexual chromosome Z, respectively. Thus, by decreasing the sequence call rate, we can expect an increase in the number of missing genotypes. However, missing genotypes are not missing for each individual, leading to a higher number of markers for each individual and for the population. These markers would be in stronger LD than those observed in cattle. These markers would be helpful to accurately impute the neighbouring markers with a different lattice between individuals and would lead to higher imputation accuracy of missing data.

Finally, a rather low loss of detected SNPs with the different RADseq methods on real data could be observed compared to the expected number of SNPs with the in silico studies.

### Imputation accuracy

The results showed an increase in mean correlations between true and imputed genotypes with an increasing number of SNPs in common with the SNP HD ship. Indeed, the mean correlation was 0.7906 with 1,797 SNPs for EcoRI, 0.9691 with 11,193 SNPs for the double digestion TaqI and PstI, and 0.9735 with 14,390 SNPs for PstI. This increase in mean correlations was consistent with results from the literature [5, 7, 8, 16]. To realize imputations, a higher number of SNPs to return to the HD genotypes resulted in an increased number of genotypes available to identify the corresponding reference haplotypes in the haplotype reference library. Thus, the probability of identifying an incorrect haplotype for the selection candidate decreased.

In addition, these results can be compared to the results presented in Herry et al. [16], where several low-density SNP chips were designed according to an equidistant methodology or a methodology based on linkage disequilibrium. Indeed, the same individuals were studied in this study as in the previous study. The results obtained with the different RADseq methods (Table 2) can be compared to the results of low-density SNP chips designed with the equidistant methodology. With EcoRI, the results were significantly lower than those obtained with a low density of 2 K SNPs. This was partly due to the difference in SNP density (216 SNPs). For very low density, a difference of a few SNPs can impact mean correlations.

With TaqI, the results were significantly higher than the results for the low-density SNP chip of 4 K SNPs. This was partly due to the difference in SNP density (103 SNPs). For AvaII, PstI, and the association of TaqI and PstI, the results were significantly greater than those obtained with an equidistant low-density SNP chip of 15 K SNPs, with fewer SNPs than on the low-density SNP chip. In detail, the equidistant methodology used in Herry et al. [16] resulted in a decrease in the mean correlation from macrochromosomes to microchromosomes. In this study, only EcoRI led to a decrease in mean correlations from macrochromosomes to microchromosomes. The other enzymes as well as the double use of TaqI and PstI led to rather stable results among chromosomes.

To understand these differences, the results need to be studied according to the type of chromosome. With the equidistant methodology used in Herry et al. [16], the SNP distribution per Mb was assumed to be the same for each type of chromosome, and a decrease in mean correlation was observed with a decrease in chromosome size. For instance, the SNP chip with 10 K equidistant SNPs (10Kequi) was assumed to have 10 SNP. Mb^− 1^ for each type of chromosome, and the mean correlations decreased with chromosome size. With EcoRI, the ratio was only 1.6 SNP. Mb^− 1^ for macro-chromosomes, 2.1 SNP. Mb^− 1^ for intermediate chromosomes and 2.3 SNP. Mb^− 1^ for microchromosomes, and the mean correlations decreased with the size of the chromosomes. However, compared to the close and expected ratio of 2 SNP. Mb^− 1^ for 2Kequi, the significantly lower results obtained with EcoRI could be explained by the distribution of the SNPs on chromosomes that were not equidistant. In contrast, with PstI, the ratio was 8.9 SNP. Mb^− 1^ for macro-chromosomes, 18.8 SNP. Mb^− 1^ for intermediate chromosomes and 42.7 SNP. Mb^− 1^ for microchromosomes, and the mean correlations were rather stable according to the type of chromosome. For 15Kequi, the ratio was expected to be approximately 15 SNP. Mb^− 1^ for each type of chromosome with a decrease in mean correlations with chromosome size. The use of PstI enabled a large increase in the number of SNPs detected on microchromosomes. Thus, in addition to the nonuniform distribution of the SNPs, there was an optimization in the number of SNPs on macrochromosomes and a densification on microchromosomes. At an equivalent SNP density, this densification on microchromosomes was even higher than that observed with SNP chips based on linkage disequilibrium. All these factors can explain the higher results obtained for enzymes enabling optimization of the number of SNPs on macrochromosomes and densification of this number on microchromosomes compared to an equidistant methodology. The use of a restriction enzyme that enabled densification of the Gallus gallus microchromosomes was thus of major importance to obtain high imputation accuracy.

The case of sexual chromosome Z was particular for each enzyme tested with a lower imputation accuracy (except for EcoRI) and a lower SNP distribution per Mb than that observed for the other type of chromosome. With the HD SNP chip, among the 26,867 SNPs of the Z chromosome, only 10,113 SNPs were informative for the line studied. Among the 10,113 SNPs, only 63, 113, 314, 316 and 360 SNPs were in common with those obtained with EcoRI, TaqI, TaqI and PstI and AvaII and PstI, respectively. However, by looking at the ratio of the number of fragments of interesting sizes per Mb (Fig. 3), the values obtained from the sexual chromosome Z were close to the ratio of macrochromosomes, except for AvaII, which was closer to the ratio of intermediate chromosomes. Thus, the lower SNP distribution per Mb was explained by a quite reduced number of SNPs in common with the SNPs of the HD SNP chip after quality control. This led to a lower imputation accuracy for chromosome Z regardless of the enzyme employed.

### Impact on genomic evaluation

#### Impact of imputation errors

For the top 150 individuals of G1 and the 67 G1 breeders, the results showed that Spearman correlations increased for all traits studied with an increase in the number of SNPs in common with the HD SNP chip from EcoRI (1,797 SNPs) to AvaII and PstI (12,453 and 14,390 SNPs, respectively). This increase in mean correlations was consistent with the literature. Aliloo et al. [7] showed in bovines that the correlations between GEBVs estimated with the HD SNP chip (Illumina BovineHD BeadChip with 777 K SNPs) and GEBVs estimated with imputed low-density SNP chip of 4,013 and 25,410 SNP were, respectively 0.9398 and 0.9927. These results can be compared with the results presented in Herry et al. [60], where several low-density SNP chips were designed according to an equidistant methodology or a methodology based on linkage disequilibrium. The same individuals were used in this study, and the impact of the use of these low-density SNP chips on genomic evaluations was evaluated. Thus, the results obtained using different RADseq approaches (Table 5) can be compared to the results of low-density SNP chips designed using the equidistant methodology.

With EcoRI, the results were lower than those obtained with a low density of 2 K SNPs for all traits studied. The differences were not significant except for ESC of the top 150 individuals, for which the correlations were 0.6481 with EcoRI and 1,797 SNPs and 0.7956 with an equidistant low-density SNP chip of 2013 SNPs. The results using TaqI were greater but not significantly different from the results obtained with an equidistant low-density SNP chip of 4 K SNPs. Finally, the results for AvaII and PstI and the association of TaqI and PstI were also greater but not significantly different from the results obtained with an equidistant low-density SNP chip of 15 K SNPs. However, the number of SNPs used with AvaII or PstI and with TaqI and PstI was lower than the 14,963 SNPs of the 15Kequi SNP chip. Thus, similar results were obtained with SNPs lower than the 15Kequi SNP chip with these two enzymes. In addition, at an equivalent SNP density, SNP chips based on linkage disequilibrium yielded lower results than those based on equidistant methodology. Except for EcoRI, this was no longer the case for the results obtained using RADseq approaches. These differences can be explained by the SNP distribution according to the type of chromosome. As seen previously, the gene density is greater on microchromosomes than on macrochromosomes [70]. Combined with the optimization of the number of SNPs on macrochromosomes and the densification on microchromosomes, this can explain why greater but not significantly different correlations were obtained with TaqI, TaqI and PstI and AvaII and PstI compared with equidistant low-density SNP chips.

#### Impact on the relative accuracy of genomic evaluation

The impact on the relative accuracy of genomic evaluations of comparing the results of the “Full_HD” genomic evaluations and genomic evaluations based on ancestry with imputed HD genotyping yielded lower results for EcoRI, except for ESC, compared to the results obtained using the other enzymes or the HD SNP chip. The differences observed in relative accuracy were not significant. The results concerning the other enzymes were closer to the maximum relative accuracy attainable with HD information, but the differences observed were not significant. These results were consistent with previous results showing a low impact of imputation errors on genomic evaluations of the 67 breeders. Indeed, except for EcoRI, Spearman correlations were greater than 0.98 for each trait studied by comparing the GEBVs based on ancestry obtained with true HD genotyping or imputed HD genotyping. These results could be different for the 150 best individuals for a specific trait. Indeed, among these 150 best individuals, only a portion are selected as breeders and have offspring. The best individuals for a specific trait are not necessarily chosen as breeders because this choice is based on a multitrait index. All these results were in agreement with the literature. Chen et al. [71] showed in bovines that the accuracy of genomic evaluation calculated with Pearson correlations between direct genomic values with true 50 K genotyping and bull proofs was 0.61 for milk yield and 0.62 for somatic cell score. With imputed 50 K genotyping from 6 K SNPs, Pearson correlations were 0.61 for milk yield and 0.62 for somatic cell score. Likewise, Herry et al. [60] showed that the use of equidistant low-density SNP chips with an SNP density higher than 2 K SNPs led to no difference in Pearson correlations compared with that obtained using the HD SNP chip. Indeed, for the 2Kequi SNP chip, the correlations were 0.4929 for EW, 0.3126 for ESC, 0.3775 for ESS and 0.4157 for AH. For the 15Kequi SNP chip, the correlations were 0.4889 for EW, 0.2574 for ESC, 0.3955 for ESS and 0.4762 for AH. There was no significant difference with the correlations obtained with the HD SNP chip.

Therefore, the use of restriction enzymes in the GGRS or ddRADseq approaches and the use of the SNPs detected in common with the SNPs of the HD SNP chip are an interesting alternative to low-density SNP chips without a decrease in the relative accuracy of genomic evaluation of the selection candidates.

### Balance between number of sequenced individuals and read depth

As seen previously, the use of different restriction enzymes led to different numbers of fragments of interest and finally a different number of SNPs detected in common with the SNPs of the high-density SNP chip. The sequencing costs depend on the number of individuals and the expected read depth of each fragment for each individual. Thus, it depends on the number of fragments to be read. In a previous study using the GGRS approach in chickens [36, 37], a sequencing depth between 5X and 7X was expected. With a ddRADseq approach, a sequencing depth higher than 7X was expected [28]. Assuming this sequencing depth, increasing the number of fragments to be sequenced would lead to a decrease in the number of individuals who could be sequenced together in a lane and finally to an increase in sequencing costs. In contrast, decreasing the number of fragments to be sequenced would lead to an increase in the number of individuals who could be sequenced together in a lane. However, a decrease in the number of fragments would result in fewer SNPs detected in common with the SNPs of the HD SNP chip. Given the heterogeneity expected between individuals with the different RADseq approaches, using EcoRI or TaqI may be too optimistic since the number of SNPs detected in common with the SNPs of the HD SNP chip would be lower than expected. On the other hand, with PstI, AvaII and the association of TaqI and PstI, given the number of fragments and the number of SNPs detected in common with the SNPs of the HD SNP chip, imputation accuracy, the results of genomic evaluation, and the expected costs of less than $50 per individual, the use of these enzymes could be an interesting alternative to low and medium density SNP chips.

Finally, the availability of sequencers with higher performance, allowing the sequencing of more individuals in a single lane of a flow cell with increasing read density, is now causing the sequencing costs for RADseq approaches to plummet. However, the cost of DNA library preparation cannot be reduced, and this step can still be expensive.

### Combining RADseq data and SNP chips for genomic selection

Among the total number of SNPs detected using the GGRS and ddRADseq methods, only 2.64% for AvaII to 3.85% for EcoRI were common to the SNPs of the HD SNP chip. However, the remaining SNPs can be useful for imputation and genomic prediction. Indeed, Brouard et al. [41] showed that using both SNP chips and GBS panels enabled better imputation accuracy for missing data than using only the GBS panel. Indeed, the addition of a SNP chip panel led to an increased number of high-quality genotypes available to identify the corresponding reference haplotypes in the haplotype reference library. This resulted in better imputation accuracy for missing data than using only the GBS panel.

Likewise, Torkamaneh and Belzile [39] focused on the imputation of untyped loci in soybeans. They showed that combining the GBS and SNP chip panel for their candidates and imputing them to a WGS level from a set of reference samples resulted in an imputation accuracy of 88.1%. With a GBS panel alone imputation accuracy was 80%. Thus, the combination of the SNP chip and GBS approach may be useful to obtain high imputation accuracy for untyped markers.

Finally, Poland et al. [38] and Elbasyoni et al. [40] dealt with this topic in depth by studying the impact of the use of GBS or SNP chip panels on genomic evaluations of wheat. Genomic prediction accuracies were assessed as the correlation between GEBVs and phenotypic values. At equivalent SNP density, they showed that significantly higher correlations were obtained for different traits with the GBS panel compared to the results obtained with the SNP chip. Increasing the number of SNPs in the GBS panel by increasing the percentage of missing data led to a correlation that was not significantly different from the results obtained with the previous GBS panel.

Thus, when applied to chickens, combining SNP chips and RADseq panels could be helpful to accurately impute missing data among RADseq datasets. However, combining SNP chips and RADseq panels to impute untyped loci would require a reference population with higher SNP density than that obtained with RADseq methods and SNP chips separately to ensure the overlap of both panels. This is currently still too expensive for routine use in a selection scheme. Finally, the use of RADseq techniques instead of SNP chips on reference and candidate populations may be an interesting approach to obtain higher genomic evaluation accuracy. However, large changes in genotyping strategies are currently needed by creating reference populations genotyped with a RADseq approach. By doing so, unmapped SNPs on the reference genome and not included on SNP chips can be used for genomic evaluations. Gains in the accuracy of genomic evaluations can be expected for traits with QTLs located in poorly known chromosomal regions.

## Conclusions

Our studies have demonstrated the value of using genome complexity reduction methods with restriction enzymes for genotyping purposes. By comparing the SNPs detected by these methods with SNPs from low-density chips with an equidistant distribution, we were able to observe a better distribution and densification of SNPs according to chromosome type. In the context of using these genotypes for genomic evaluation, the GEBVs from imputed RADseq genotypes compared to those obtained with HD genotypes show very high correlations, suggesting an interesting alternative to low density SNP chips.

## Data Availability

Data is deposited in the European Nucleotide Archive (ENA) with the accession number PRJEB58821.
